# Enhancement of Fluorescence-Based Sandwich Immunoassay Using Multilayered Microplates Modified with Plasma-Polymerized Films

**DOI:** 10.3390/s17010037

**Published:** 2016-12-25

**Authors:** Kazuyoshi Yano, Akira Iwasaki

**Affiliations:** School of Bioscience and Biotechnology, Tokyo University of Technology, 1404-1 Katakura, Hachioji, Tokyo 192-0982, Japan; iwasaki@material-sys.com

**Keywords:** microplate, plasma polymerization, sandwich-immunoassay, fluorescence enhancement

## Abstract

A functional modification of the surface of a 96-well microplate coupled with a thin layer deposition technique is demonstrated for enhanced fluorescence-based sandwich immunoassays. The plasma polymerization technique enabling the deposition of organic thin films was employed for the modification of the well surface of a microplate. A silver layer and a plasma-polymerized film were consecutively deposited on the microplate as a metal mirror and the optical interference layer, respectively. When Cy3-labeled antibody was applied to the wells of the resulting multilayered microplate without any immobilization step, greatly enhanced fluorescence was observed compared with that obtained with the unmodified one. The same effect could be also exhibited for an immunoassay targeting antigen directly adsorbed on the multilayered microplate. Furthermore, a sandwich immunoassay for the detection of interleukin 2 (IL-2) was performed with the multilayered microplates, resulting in specific and 88-fold–enhanced fluorescence detection.

## 1. Introduction

The fluorescence-based immunoassay is widely used in biotechnology and clinical testing. Antibodies or antigenic proteins can be easily labeled with a variety of fluorophores for fluorescence detection. Therefore, improving the fluorescence sensitivity and lowering the detection limit of targets is highly critical, especially for the early diagnosis of diseases. Strategies which are used to enhance the fluorescence intensity include applications of an environmentally sensitive dye [1] and ZnO nanostructure–modified microfluidic devices [2]. Recently, a number of sensitive bioassays based on plasmonic fluorescence enhancement using metal nanoparticles have been reported [3,4]. The increased fluorescence intensity can be attributed to an optical phenomenon called metal-enhanced fluorescence (MEF) induced by localized surface plasmon resonance (LSPR) on metal nanoparticles. Because of its great potential, MEF has been applied for highly sensitive immunoassays [5] and demonstrated 14.3- [6] and 19-fold [7] fluorescence enhancement. An ultrasensitive time-resolved fluorescence immunoassay was also achieved by coupling gold nanoparticles and a europium(III)-labeled tracer [8].

A multilayered glass slide modified with a plane metal mirror coated with an optical interference layer is a simple and promising tool for fluorescence enhancement. The multilayered substrate fabricated with approximately 100-nm-thick dielectric LiF as an optical interference layer on the surface of a silver mirror can exhibit up to 400-fold enhancement of fluorescence for Rhodamine B compared with a bare glass slide [9,10]. Other dielectric materials instead of LiF, such as SiO_2_ [11] and Al_2_O_3_ [12,13], have also been utilized for the optical interference layers. Those modified substrates have been applied in many bioassays [14,15,16]. This enhancement effect could be attributed to the double interference of both the excitation light and fluorescence in the optical interference layer, whereas an electric field oscillating parallel to the substrate might also be partly involved [12]. It was revealed that the change in the fluorescence enhancement had more than one peak and valley in the wide range of thicknesses of the optical interference layers [10,12]. Interestingly, those peaks and valleys were found to appear periodically, which indicates that the enhancement effect is based on the interference phenomenon and highly dependent on the thickness of the optical interference layer.

We have recently focused on plasma-polymerized films (PPFs) as excellent alternatives to SiO_2_ or Al_2_O_3_ for multilayered substrates. Plasma polymerization is a technique to deposit organic thin films on substrates in a glow discharge under a monomer vapor phase [17,18]. PPFs have highly branched and cross-linked, and thus pin-hole–free, polymer network structures. PPFs are chemically and mechanically stable and provide good adhesion to many materials including glass, silicon and plastic substrates while retaining their bulk properties. PPFs have been utilized as protective [19] or gas-selective [20] membranes, and much more widely applied in biological and bioanalytical fields, such as in cell biology [21], enzyme sensors [22,23], immunosensors [24,25], single-nucleotide polymorphism (SNP) typings [26,27] and capillary electrophoresis [28]. It was also demonstrated that protein-embedded PPFs deposited on glass slides could function without any loss in biological activity from direct exposure to plasma to create DNA [29,30] and antibody [25] arrays. Hexamethyldisiloxane (HMDS) is one of the widely used monomers of PPFs because the chemical structures of plasma-polymerized HMDS have been well characterized by spectroscopic analyses such as infrared (IR) and X-ray photoelectron spectra (XPS) measurements [31,32]. We have previously demonstrated that PPFs using HMDS as a monomer could serve as optical interference layers of the multilayered glass slides and that contributed up to 81-fold fluorescence enhancement in immunoassays [33].

**Figure 1:**
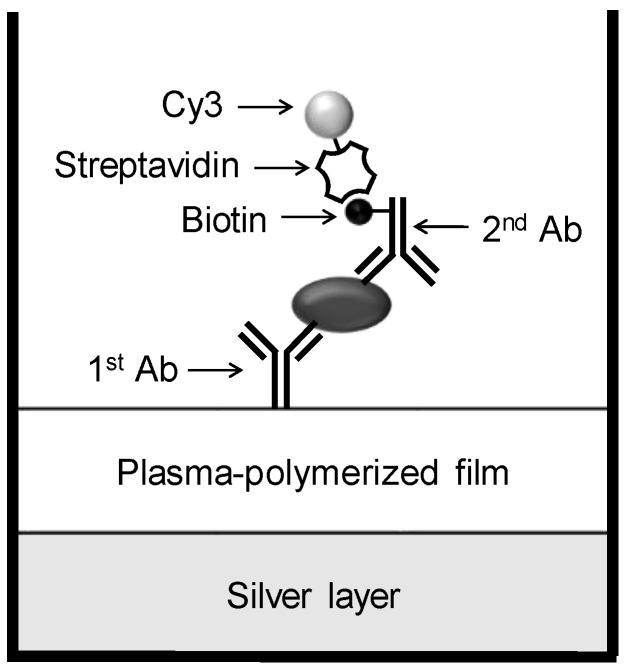
Schematic diagram of a sensitive sandwich immunoassay using a multilayered microplate.

In this study, we extend the multilayered structures modified with PPFs to a more versatile format, that is, a 96-well plastic microplate. Microplates are the most standard and common formats used in basic bioassays and clinical tests, where sensitive detection of target molecules is always of great interest. A number of functionally modified microplates are already commercially available, e.g., ones modified with streptavidin or amino group. There are also some reports regarding further functionalization of microplates by coating with molecularly-imprinted polymers [34] or metal oxide [35]. However, most of them have been prepared for assisting the effective immobilization of biomaterials such as antibodies or oligonucleotides, not for enhancing fluorescence signals. Our simple approach for sensitive sandwich immunoassays is depicted in Figure 1. The surface of the 96-well microplate is modified with a silver layer and PPF to obtain the functional multilayer structure. After immobilization of the primary antibody on the PPF, the antigen, the secondary antibody and the Cy3-labeled protein are consecutively added to allow specific interactions and enhancement of the fluorescence intensity.

## 2. Materials and Methods

### 2.1. Materials

HMDS was purchased from Shin-Etsu Chemical Co. Ltd. (Tokyo, Japan). Goat-derived Cy3-labeled anti-mouse IgG antibody and Cy3-labeled streptavidin were from GE Healthcare Bio-Sciences Corp. (Piscataway, NJ, USA). Mouse IgG and rabbit IgG were purchased from Sigma-Aldrich, Inc. (St. Louis, MO, USA). Interleukin 2 (IL-2) Human Antibody Pair including anti-IL-2 antibody and biotin-labeled anti-human IL-2 antibody was purchased from Invitrogen Corp. (Camarillo, CA, USA). Other reagents were of analytical grade from Wako Pure Chemical Industries, Ltd. (Osaka, Japan). Black 96-well polystyrene microplates with flat bottoms consisting of six 16-well modules (F16 MaxiSorp, FluoroNunc) were purchased from Thermo Fisher Scientific Inc. (Waltham, MA, USA).

### 2.2. Deposition of Metal Layers

Metal layers were deposited onto 16-well modules of the microplates by a sputtering apparatus (CFS-4ES, Shibaura Mechatronics Corp., Kanagawa, Japan). For preparation of a calibration curve, silver layers with various thicknesses as the metal mirror were formed onto microplates by changing deposition time. Wells of conventional microplates are too deep for any apparatus to directly measure the thickness of the layers formed on the bottom. Therefore, the bottom plate and the upper horizontal well part were cut off mechanically, and the thickness of deposited layer on the well surface was measured with a surface profile meter (Dektak8, Veeco Instruments, Inc., Plainview, NY, USA). Glass slides (76 × 26 mm^2^, Matsunami Glass Ind. Ltd., Osaka, Japan) were also used and modified similarly as control formats. For preparation of multilayer structures on glass slides or microplates for enhanced immunoassays, a 15-nm-thick chromium layer as an adhesive and a 200-nm-thick silver layer was deposited successively onto those formats.

### 2.3. Preparations of PPFs

PPFs were prepared using a plasma deposition system (Model BP-1, Samco Inc., Kyoto, Japan) with two external electrodes set along the vacuum chamber horizontally, 15 cm above the sample stage as reported previously [29,33]. A radio frequency (RF) generator (Model RFG-300, Samco Inc., Kyoto, Japan), coupled to a matching box to minimize reflected power, was also employed. The working frequency of the power supply was 13.56 MHz.

A 16-well module of the microplate was placed on the sample stage of the plasma deposition apparatus. The chamber was evacuated, and a carrier gas of HMDS was added to the chamber at a constant flow rate of 20 ccm. The back ground pressure was approximately 0.4 Torr. Then plasma was discharged at an RF power of 100 W to deposit the PPF on the microplate. To obtain a calibration curve, a number of PPFs of different thicknesses were prepared by varying the deposition time. The thickness of each PPF deposited on the surface of the well bottom was measured similarly to that of metal layers using the surface profile meter. Glass slides were coated with PPFs in the same manner as control formats.

A multilayer structure for fluorescence-based enhanced immunoassay was similarly prepared on a microplate coated with a 200-nm-thick silver layer. PPF deposition time was set according to the calibration curve, whereas the deposition was analyzed every time by measuring the thickness of PPFs simultaneously formed on different reference microplates placed on the same sample stage.

### 2.4. Homogeneous Fluorescence Detection of Cy3-Labeled Antibody in the Multilayered Microplate

Cy3-labeled anti-mouse IgG antibody was dissolved and diluted in phosphate-buffered saline (PBS, pH 7.4) to obtain 1.6 g/mL solution. Then each 100 L of the solution was applied to three wells of a multilayered microplate with a 200-nm-thick silver layer and 53-nm-thick PPF, and of an unmodified one. Immediately a two-dimensional Cy3 fluorescence image of microplates was obtained using 532 nm excitation with a bandpass filter of 605 nm of a scanner (Pharos FX, Bio-Rad Laboratories, Inc., Hercules, CA, USA). Fluorescence intensity was quantified as follows: an empty circle equivalent to the same area of a well of microplate was created on the scanned image by Quantity One Software (Bio-Rad). Circles were copied and overlaid on all the wells of interests, and fluorescence intensities within the circle area were calculated by subtracting the background signals from wells containing only buffers on each type of microplate.

### 2.5. Fluorescence-Based Immunoassay Targeting Antigen Directly Adsorbed on the Multilayered Microplate

Mouse IgG and rabbit IgG were dissolved in PBS to obtain 1 mg/mL solutions and further diluted to desired concentrations. IgG samples of 100 μL were applied to wells of a multilayered and an unmodified microplates and incubated for 2 h for physical adsorption. After discarding the IgG solutions, the wells were washed with 300 μL of PBS including 0.1% Tween 20 (PBS-T) three times, blocked by 200 μL of blocking solution [20 mM HEPES (pH 7.4), 137 mM NaCl, 30 mM KCl, 5 mM MgCl_2_, 0.1% Tween 20, and 1 mg/mL human serum albumin (HSA)] for 2 h and washed with PBS-T three times. Cy3-labeled anti-mouse IgG antibody (100 μL of 10 μg/mL solution) was applied to the wells, incubated for 30 min and washed with PBS-T 3 times. Finally, 150 μL of PBS-T were applied and a two-dimensional fluorescence image was recorded by the scanner. The fluorescence intensity from each well was calculated by subtracting the background signals of PBS-T applied to wells of the multilayered or unmodified microplates. All immunoassay procedures were performed at room temperature.

### 2.6. Sandwich Immunoassay of IL-2 on the Multilayered Microplates

IL-2 detection was performed by sandwich-immunoassay on multilayered and unmodified microplates. Reagents of anti-human IL-2 antibody and biotin-labeled anti-human IL-2 antibody were diluted with PBS to obtain 4 and 10 μg/mL primary and secondary antibody solutions, respectively. The primary antibody of 100 μL were applied to wells of ayered and unmodified microplates and incubated for 2 h. After discarding the solutions, the wells were washed with 300 μL of PBS-T 3 times, blocked by 200 μL of blocking solution for 2 h and washed with PBS-T three times. IL-2 solutions of 100 μL diluted to desired concentrations were applied to the wells, immediately followed by addition of the secondary antibody of 50 μL, mixed by pipetting, incubated for 1 h and washed with PBS-T three times. Cy3-labeled streptavidin (100 μL of 10 μg/mL PBS solution) was applied to the wells, incubated for 30 min and washed. After addition of 150 μL of PBS-T a two-dimensional fluorescence image was recorded by the scanner, and the fluorescence intensity was analyzed. Bovine serum albumin (BSA) was used similarly instead of IL-2 as the negative control. All procedures were performed at room temperature.

## 3. Results and Discussion

### 3.1. Calibration Curves for Thin Layer Deposition on the Surface of Microplates

**Figure 2:**
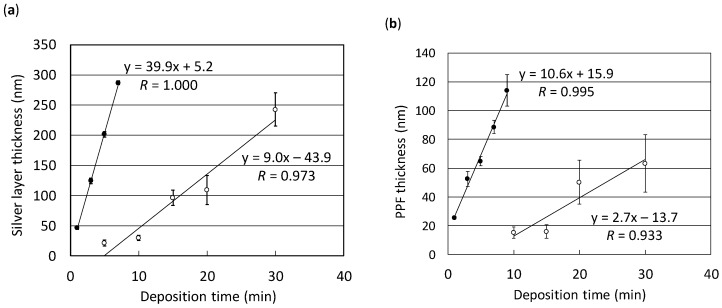
Calibration curves for silver layer (**a**) or PPF (**b**) thickness as a function of deposition time obtained with glass slides (**black circles**) and microplates (**white circles**). Error bars indicate the standard deviation of five measurements.

It was highly possible that the deposition rate for sputtering or plasma polymerization on the well surface of a microplate was different from that of a planar glass slide due to its deep well structure. Therefore, the relationship between the thickness of the silver layer or PPF and the deposition time on the microplate was examined and compared to that obtained with the glass slide. Figure 2 shows two calibration curves obtained with the sputtering apparatus (a) and the plasma deposition system (b). It was demonstrated that the thickness had a linear relationship with the deposition time even on the microplates (correlation coefficient (*R*) of 0.973 and 0.933 for the silver layer and PPF, respectively) as well as on the glass slides (*R* of 1.000 and 0.995 for silver layer and PPF, respectively). However, the deposition rates for the silver layer were approximately 40 and 9.0 nm/min on the glass slide and the microplate, respectively. Also, the rates for PPF were approximately 11 and 2.7 nm/min on the glass slide and the microplate, respectively. Decreased deposition rates on the microplates to approximately 25% of those obtained with the glass slides are considered to be due to the deep well structure of the microplate. Relatively large errors found in the calibration curves with the microplates may result from the disassembling process of the microplate for the thickness measurement as described in the Materials and Methods section.

**Figure 3:**
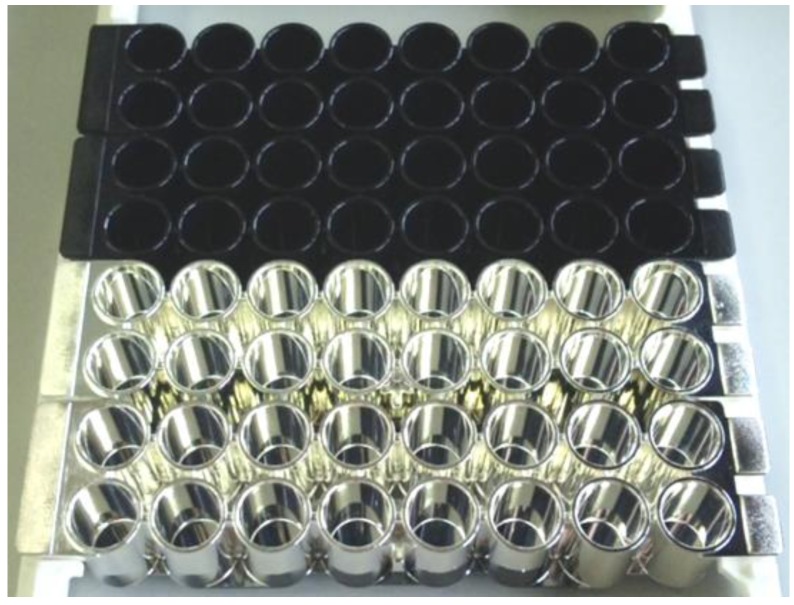
Modules of unmodified (upper) and multilayered (lower) microplates modified with a 200-nm-thick silver layer and coated with a 53-nm-thick HMDS PPF.

The appearance of the multilayered microplate is shown in Figure 3. It looks like a microplate made of metal because of the presence of the silver layer coated with transparent HMDS PPF. Through the deposition experiments it was shown that a polystyrene microplate was durable in the vacuum chambers during deposition by sputtering and plasma polymerization. It is also of significance that the PPF modification was possible for a complicated geometry such as that of a microplate with a deep well structure, which is one of the advantages of the plasma polymerization technique.

### 3.2. Homogeneous Fluorescence Detection of Cy3-Labeled Antibody in the Multilayered Microplate

**Figure 4:**
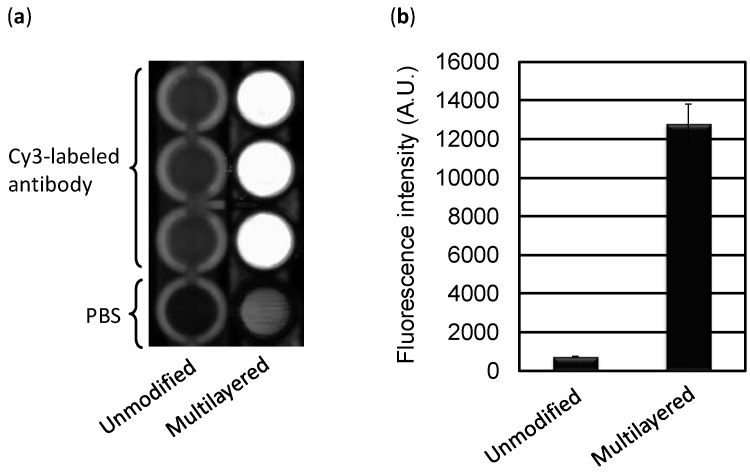
Enhancement of fluorescence intensity from Cy3-labeled antibody on the multilayered microplate. (**a**) Two-dimensional fluorescence image of the unmodified and multilayered microplate. PBS was added to wells of both microplates as a negative control; (**b**) Comparison of fluorescence signals. Error bars indicate the standard deviation of three measurements.

Before conducting the immunoassay, the basic performance of a multilayered microplate was examined by homogeneous fluorescence detection. A microplate for enzyme-linked immunosorbent assay (ELISA) was modified with a 200-nm-thick Ag layer and coated with a 53-nm-thick HMDS PPF. In our previous study the maximum fluorescence enhancement was obtained when the PPF thickness on a glass slide was 63 nm [33]. However, it was also confirmed that almost the same degree of the enhancement effect could be observed between approximately 50–70 nm. Cy3-labeled anti-mouse IgG antibody (100 μL of 1.6 μg/mL solution) was applied to the multilayered and unmodified microplates, followed by immediate fluorescence measurement by the scanner. Figure 4a shows the greatly enhanced fluorescence observed from the multilayered microplate without any immobilization step of the fluorophore-labeled protein, while the fluorescence signal was small from the unmodified microplate. This enhancement may be attributed to the fluorophore-labeled protein located approximately 60 nm above the surface of PPF. The fluorescence intensity from the multilayered microplate was approximately 18-fold compared with that from the unmodified one (Figure 4b).

Akimoto and co-workers demonstrated that a maximum 200-fold enhancement could be achieved with Al_2_O_3_ as the optical interference layer on the glass slide [12]. In this case, however, the analyte was spin-coated Rhodamine B, whereas Cy3-labeled antibody solution in PBS was applied in the modified microplate in this study. Therefore, the presence of a bulkier protein moiety in the labeled molecule might affect the enhancement effect. They also characterized the performance of the multilayered glass slide by using a carefully arranged optical measurement system including a cooled charge-coupled device as a sensitive detector and an excitation light source with which the incident angle could be adjusted and optimized. An enhancement factor of 18-fold in this study seems acceptable considering the measurement system using a general fluorescence scanner without any immobilization step of the labeled protein.

### 3.3. Fluorescence-Based Immunoassay Targeting Antigen Directly Adsorbed on the Multilayered Microplate

**Figure 5:**
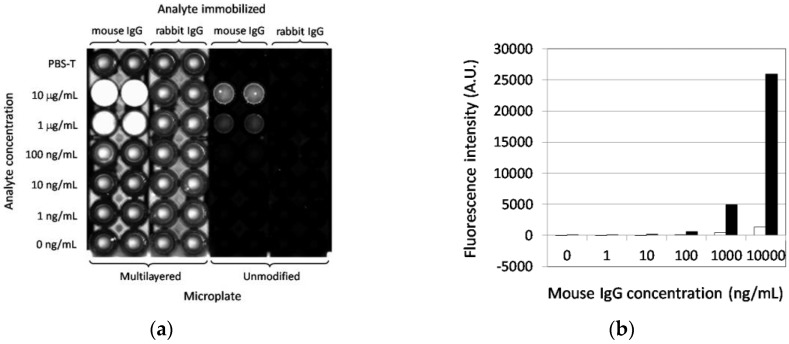
Effect of the multilayer structure on the immunoassay. Antigen-antibody interaction was evaluated using Cy3-labeled anti-mouse IgG antibody on the unmodified or multilayered microplate. (**a**) Two-dimensional fluorescence image of microplates scanned after immunoreaction; (**b**) Fluorescence intensity as a function of mouse IgG concentration obtained with the unmodified (white bars) and multilayered (black bars) microplates. Averages of two measurements are demonstrated.

As the multilayered microplate was shown to be effective for the enhancement of the fluorescence signal from the fluorophore-labeled protein without any immobilization step, it was then used for an immunoassay targeting antigen directly immobilized on the well surface. The polystyrene microplate used in this study is the “MaxiSorp” type, by which maximum adsorption of antibodies on the well surface can be expected for immunoassays. Therefore, employment of this type of microplate without any modification would be a good control. Mouse IgG and rabbit IgG were physically adsorbed to the wells of multilayered and unmodified microplates and incubated with Cy3-labeled anti-mouse IgG antibody. Figure 5a shows a two-dimensional fluorescence image of those microplates after the immunoassay. It was clearly demonstrated that the fluorescence signal from the mouse IgG was highly enhanced from the multilayered microplate, whereas the fluorescence intensity from the unmodified microplate was quite low or undetectable. The signal from the rabbit IgG was as low as that from the PBS-T sample as a reference even on the multilayered microplate, indicating specific enhancement only by the multilayer structure. A relatively high background fluorescence from the multilayered microplate was caused not only by the simple mirror effect of the silver layer but by adjusting and raising the sensitivity of the scanner so that the very low signal from the mouse IgG on the unmodified microplate could be visualized and quantified.

The fluorescence intensity of the mouse IgG samples from the unmodified and multilayered microplates is summarized in Figure 5b. An enhanced and concentration-dependent signal on the multilayered microplate is shown, and the enhancement factor was 19-fold at 10 μg/mL antigen compared with that on the unmodified microplate. Moreover, 100 ng/mL of antigen could be detected on the multilayered microplate, while for the unmodified one, 1000 ng/mL was the lowest detection limit, indicating an approximately 10-fold enhancement of sensitivity contributed by the multilayer structure. The results also imply that the enhancement effect could be obtained by direct immobilization of antigens on the surface of the PPF as well as by homogeneous detection. An enhancement factor of 10 is greater than that of approximately five obtained with an environmentally sensitive dye [1], but lower than the approximately 60 obtained with a mirror slide coated with Al_2_O_3_ as the optical interference layer [15]. However, the assay in the latter was based on binding between biotin and Cy3-labeled streptavidin, the affinity of which is much greater than that of antigen-antibody reactions.

In our previous study, the effects of types of modification were compared by preparing four glass slides: unmodified, modified with a silver layer only, modified with an HMDS PPF only, and modified with an HMDS PPF on a silver layer [33]. Fluorescence-based immunoassays performed on those substrates under the same conditions revealed that the fluorescence signal was highly enhanced on the multilayered substrate, whereas the fluorescence intensities from three other control substrates were quite low or undetectable. The result indicates that both layers of silver and PPF were necessary for a large enhancement and that the contribution of PPF as a simple adsorption support for biomaterials was very much limited, which could be also applicable to this study.

### 3.4. Sandwich Immunoassay of IL-2 on the Multilayered Microplates

**Figure 6:**
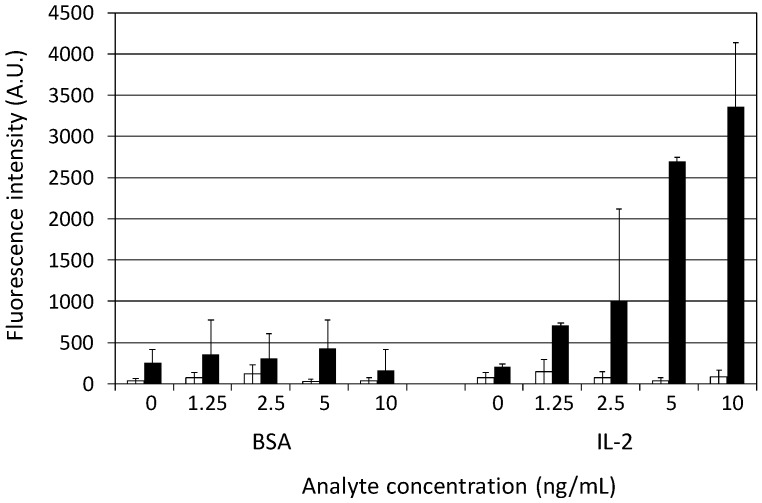
Sandwich immunoassay targeting IL-2 detected by using Cy3-labeled streptavidin on the unmodified (white bars) or multilayered (black bars) microplate. Error bars indicate the standard deviation of three measurements.

Conventional immunoassays utilize a pair of two antibodies which bind different epitopes of the antigens for sandwich detection. To widen the applications of the multilayered microplate, therefore, we further evaluated its efficacy for the sandwich immunoassay by using human IL-2 as a model target. Anti-human IL-2 antibody and biotin-labeled anti-human IL-2 antibody for the sandwich immunoassay were commercially available and used as the primary and secondary antibody, respectively. As shown in Figure 6, a highly enhanced fluorescence intensity was observed from the IL-2 samples on the multilayered microplate. The detection of 1.25 ng/mL IL-2 could be achieved for the multilayered microplate, whereas the detection limit was higher than 10 ng/mL for the unmodified one. The enhancement factor was 88-fold at 5 ng/mL IL-2 compared with that on the unmodified microplate. The fluorescence intensity for the BSA concentration of 0–10 ng/mL was quite low, the same as that for 0 ng/mL IL-2, indicating that the sandwich immunoassay with the multilayered microplate was not only sensitive but specific.

Park and co-workers demonstrated the detection of cytokines at a subnanogram per milliliter level by a fluorescence-based sandwich immunoassay [36]. They prepared the protein G–terminated glass substrate so that the orientation of the immobilized antibody could be controlled. In our study, the primary antibody was immobilized on the PPF by a simple physical adsorption method, which might lead to a random and improper orientation. We have previously demonstrated that streptavidin and antibody could be embedded in PPFs without any loss of their biological activities to create DNA [29,30] and antibody [25] arrays, respectively. Therefore, it is highly possible to perform more sensitive detection by embedding protein G or protein A in PPFs for the immobilization of antibodies with proper orientation. The hydrophobicity of PPFs may also affect the enhancement of the fluorescence intensity. A number of organic chemicals other than HMDS have been utilized as monomers of plasma polymerization for sensor applications, e.g., acetonitrile [23], ethylenediamine [24] and di(ethylene glycol) mono vinyl ether [30], none of which have been characterized for application in optical interference layers. HMDS PPF has hydrophobic properties [19,20], and other films prepared with monomers such as allylamine show hydrophilic natures [18]. Therefore, for increasing the amount of immobilized biomolecules or avoiding nonspecific adsorption, monomers could be optimized depending on molecular recognition elements or analytes in terms of more sensitive detection.

This study demonstrates the potential of PPFs for fluorescence enhancement in sandwich immunoassays. The plasma polymerization technique is based on simple batch processing without any precise design or control of the size, shape and density of the surface structure such as in MEF-based sensing systems. One of the important aspects of plasma polymerization is that modification by this technique is suitable not only for plane materials such as glass slides but also for those with complicated geometries, such as deep holes, trenches and microgrooves. To our knowledge, this is the first report to apply the plasma polymerization technique to modify the well structure of microplates and further to apply it to the enhanced fluorescence-based sandwich immunoassays. As PPFs have already been used for many bioanalytical fields, it will be further applied to sensing devices for highly sensitive detection coupled with the microplate format.
